# Evaluation of functional methods of joint centre determination for quasi-planar movement

**DOI:** 10.1371/journal.pone.0210807

**Published:** 2019-01-17

**Authors:** Lin Meng, Craig Childs, Arjan Buis

**Affiliations:** 1 Department of Biomedical Engineering, University of Strathclyde, Glasgow, United Kingdom; 2 Tianjin International Joint Research Center for Neural Engineering, Academy of Medical Engineering and Translational Medicine, Tianjin University, Tianjin, China; University of Rome, ITALY

## Abstract

Functional methods identify joint centres as the centre of rotation (CoR) of two adjacent movements during an ad-hoc movement. The methods have been used for functionally determining hip joint centre in gait analysis and have revealed advantages compared to predictive regression techniques. However, the current implementation of functional methods hinders its application in clinical use when subjects have difficulties performing multi-plane movements over the required range. In this study, we systematically investigated whether functional methods can be used to localise the CoR during a quasi-planar movement. The effects of the following factors were analysed: the algorithms, the range and speed of the movement, marker cluster location, marker cluster size and distance to the joint centre. A mechanical linkage was used in our study to isolate the factors of interest and give insight to variation in implementation of functional methods. Our results showed the algorithms and cluster locations significantly affected the estimate results. For all algorithms, a significantly positive relationship between CoR errors and the distance of proximal cluster coordinate location to the joint centre along the medial-lateral direction was observed while the distal marker clusters were best located as close as possible to the joint centre. By optimising the analytical and experimental factors, the transformation algorithms achieved a root mean square error (RMSE) of 5.3 mm while the sphere fitting methods yielded the best estimation with an RMSE of 2.6 mm. The transformation algorithms performed better in presence of random noise and simulated soft tissue artefacts.

## Introduction

Gait analysis is useful for understanding pathological movement patterns and evaluating efficiency of therapeutic interventions. Model predictions of joint kinematics have demonstrated particular sensitivity to the location of the joint centres [1]. Errors in joint centre localisation will lead to substantial inaccuracies in kinematic and kinetic calculations and thus affect assessor interpretation. Traditional gait models determine the joint centres based on the location of markers at identifiable anatomical landmarks. The precision of landmark identification replies on user experience, knowledge and expertise. Cappozzo [2] proposed a functional approach for hip joint centre (HJC) localisation in which a fixed centre of rotation (CoR) between the femur and pelvis is determined from relative rotation of the two segments. The method showed the potential for reducing reliance on anatomical landmarks in traditional gait models.

Attempts have been made to evaluate the accuracy of functional methods for localising the HJC in simulation [3–5], in-vitro [6] and in-vivo human studies [7–9]. The use of simulation approaches provides practical guidelines for the use of functional methods in human subjects [5, 10]. Camomilla et al. [5] suggested that star-arc movement may be preferred to achieve an optimal estimation with a range of movement greater than 30 degrees, which has been widely adapted in human studies. The superiority of functional methods over predictive regression methods for HJC estimation have been proven in in-vivo human studies [9, 11, 12]. Functional methods achieved better agreement with the HJC measured by dual fluoroscopy (11.0 ± 3.3 mm) than predictive methods (18.1 mm ± 9.5 mm) in the study with able-bodied subjects [9]. The accuracy of functional methods could be affected by reduced range of movement (RoM) [13, 14], number of cycles [4], algorithms [14] and marker locations [8]. The accuracy of HJC localisation replies upon the nature of hip motion [4]. Piazza et al. [7] demonstrated that the planar hip motion achieved the worst HJC estimation in human subject (∼ 70 mm) and concluded that the motion pattern is important to functionally determine the HJC. A complete exploration of all degrees of freedom of the hip joint would improve the functional method performance [15]. The requirement of specific hip motion limits the application of functional methods on subjects with restricted plane of motion.

A couple of mechanical simulation studies have shown that functional methods achieved a good HJC estimation when the movement was restricted in the sagittal plane. Piazza et al. [10] demonstrated that the planar hip motion with a range of 30 degrees does not significantly affect HJC location accuracy. Siston et al. [16] reported similar results that the functional method obtained CoR estimation with a mean error of 4.3 ± 1.3 mm for the same hip movement. It needs to be noted that, with purely planar motion, the algorithms are theoretically not able to determine the HJC in the direction that is perpendicular to the plane of motion. Siston et al. [16] explained that they was able to achieve unique solutions with both algorithms due to some out-of-plane motion. This suggests that functional methods can determine the HJC from quasi-single planar movement. The question then arises as to what factors affect the performance of the HJC estimation with the sagittal hip movement resulting in worst HJC estimation in human subjects [7]. To the authors’ knowledge, factors that influence the accuracy of HJC localisation have not been systematically studied for this particular scenario. A thorough study would help to improve the protocol for HJC determination and make functional methods more suitable for subjects with motor impairments.

This paper aims to gain a thorough understanding of how the implementation of functional methods affects the CoR estimation during a quasi-planar movement. With a mechanical linkage on which the joint centre can be easily measured, various factors were investigated including functional methods, range of movement, speed of movement, marker cluster location, marker cluster size and distance to the joint. The effects of local coordinate system variation and marker placement on the CoR estimation were evaluated with four different functional methods and with introduction of noise in marker trajectories. The results were compared and analysed in order to generalise the conclusions for the application of functional methods.

## Materials and methods

**Fig 1:**
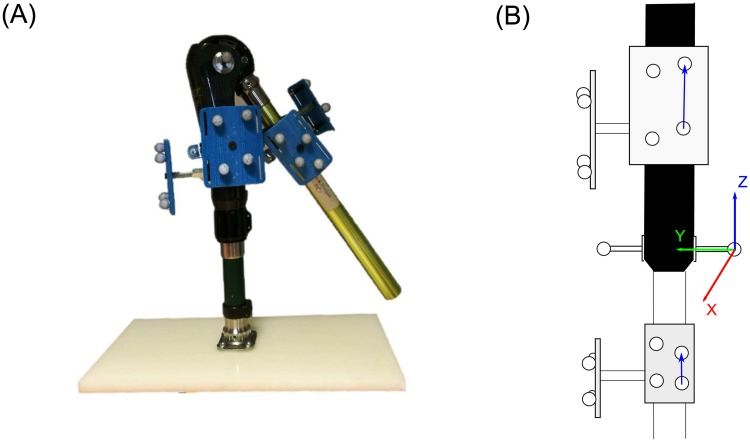
The mechanical linkage with a hinge joint. (A) The proximal segment was mounted on stand and remained static during movement while the distal segment was rotated manually about the joint. Marker clusters were attach to the front and lateral sides of both segments. The joint centre was determined using two markers placed on the two sides of the joint. (B) Joint coordinate systems were originated at the medial marker. Medial-lateral (y) axis was defined as a unit vector pointed to lateral joint marker from medial marker, inferior-superior (z) axis was perpendicular to y in the frontal plane of the corresponding segment in a superior direction and x axis was cross product of y and z axes.

We conducted a series of experiments using two rigid segments that were connected with a hinge joint as shown in Fig 1. The proximal segment was mounted on a stand and remained static while the distal segment was rotated manually about the joint so that a quasi-planar movement can be produced. Marker clusters were constructed with four retro-reflective markers (14 mm diameter) attached to a rigid plate. Two sizes of rigid marker clusters were used. Large clusters were mounted to the front and lateral side of the proximal segment while the small ones were attached on the front and lateral side of the distal segment (Fig 1). A 12-camera Vicon motion analysis system (Oxford Metrics; UK) was used to record three-dimensional marker trajectories with a capture frequency of 100 Hz. All cameras were located 3∼5m away from the object.

The distal segment was moved in four motion patterns with different velocities and ranges of motion: slow normal, slow excessive, fast normal and fast excessive respectively (Table 1). A rod attached to the proximal segment was used to limit range of motion to 45 degrees (normal) and 90 degrees (Excessive) by adjusting the rod’s height. Motion velocity was approximately controlled by manual timing. Each motion pattern was recorded for a duration of 30 seconds and repeated 5 times. These motions were repeated when the cluster size and distance were changed (Table 1).

**Table 1:** Description of experimental and analysis factors.

Factors	Description	
Range of movement	Normal	Flex 45 degrees, then extend to 0 degree
	Excessive	Flex 90 degrees, then extend to 0 degree
Speed of movement	Slow	1 cycle approximately 5 seconds
	Fast	1 cycle approximately 2 seconds
Cluster placement	L	Lateral side of the segment
	F	Front side of the segment
Cluster distance	d1	Distance between the centroid of marker cluster to CoR is 25cm
	d2	Distance between the centroid of marker cluster to CoR is 10cm
Cluster size	Large	Mean marker distance is approximately 6.5 mm
	Small	Mean marker distance is approximately 4.5 mm
Functional methods	ASF	Algebraic sphere fitting with bias compensation
	GSF	Geometric sphere fitting
	CTT	Centre transformation technique
	SCoRE	Symmetrical centre of rotation estimation

Four formal methods were analysed. Two methods belonged to sphere fitting family, geometric sphere fitting (GSF) [2, 3] and algebraic sphere fitting (ASF) with bias compensation [17, 18], and two belonged to coordinate transformation methods, centre transformation technique (CTT) [7] and symmetric centre of rotation estimation (SCoRE) [3]. Local coordinate systems were created using three markers of each cluster to compute 4×4 matrices representing transformation of the two segments. In CTT and SCoRE, the homogeneous transformation between the proximal and distal segments was calculated and the CoR was determined using these transformations during a motion trial. For the sphere fitting methods, after marker positions on the distal clusters were transferred to the corresponding proximal local coordinate, the CoR was located by fitting a sphere to marker positions using least square optimisation methods. Five cycles of movement were included to compute the CoR. As one cluster on each segment was selected, there were four different cluster placement combinations (LL, LF, FL, and FF).

One method, one motion pattern, one cluster placement, one cluster size and one cluster distance were chosen successively with different modalities. The combination of these characteristics resulted in 128 different tests. All computation was performed using Matlab 2017a (MathWorks, Natick, MA, USA). The joint centre (*c*) was determined as the middle point of two markers that were attached to the sides of the joint (Fig 1). The CoR location (*c*_*est*_) was estimated for each test. Root mean square error (RMSE) between the estimated CoR (*c*_*est*_) and reference CoR locations (*c*) in all frames were calculated following Eq 1.

$$RMSE=\sqrt{\frac{\sum_{i=1}^{N}{∥c_{est}\left(i\right)-c\left(i\right)∥}^{2}}{N}}$$ (1)

where *N* is the total frame number.

All tests were analysed through a general linear model including analysis of variance (ANOVA) with the following factors: cluster placement, range of movement, speed of movement, functional methods, cluster distance and cluster size. Bonferroni simultaneous tests and grouping analysis were performed in order to further investigate the differences in modalities at *α* < 0.01.

To investigate the effect of local coordinate location on the CoR estimation, the relationship between the origin and CoR location error was evaluated. Joint coordinate systems were created as shown in Fig 1B. The origin of the proximal coordinate system was varied with increment steps of 10 mm: along x axis (from 50 to 100 mm), y axis (from 0 to 150 mm) and z axis (from 0 to 200 mm). Functional CoR was computed by the four different methods when the proximal coordinate location varied. The variation of the distal coordinate origin lays in an area where the values on x axis ran from -50 to 100 mm, the y axis values ran from 0 to 150 mm and the z values in the range between -200 and 0 mm with a step of 10 mm increment. The CTT and SCoRE computed the CoR using new transformations after shifting the origin of the distal coordinate. As the sphere fitting algorithms require marker trajectories on the distal segment, the distance of the marker centroid to the joint centre was studied instead. A set of markers was randomly selected from eight markers of the front and lateral distal clusters. The marker selection follows two prerequisites: 1) at least three markers were chosen; 2) at least one marker was selected from each cluster. 208 marker combinations were available and their corresponding distances from the marker centroid to the joint centre were calculated. Distance values were ordered from the lowest to the highest and then assigned to a normalised weight. A Pearson’s correlation coefficient was used to determine the correlation between the proximal/distal coordinate/markers location and RMSE results with a significant level set to 0.05.

The noise was introduced to simulate soft tissue artefacts (STA) affecting marker trajectories and the sensitivity of functional methods was tested. In the first part, random Gaussian noise of varying amplitude (5, 10, 15 and 20 mm) was introduced as noise source in our study. A study showed that over 90% of measured STA components is in the frequency range of 0 ∼ 10 Hz [19]. A fourth-order low-pass Butterworth filter with a cut-off frequency of 10 Hz was used to remove redundant high frequency noise. Studies have shown that the STA depends on the segment area on which a particular marker is located, the subject anthropometry, and the type of the activity [20, 21]. Due to the limitation of Gaussian noise modelling, STA modelled from a real human subject was also considered in the study. The components of STA consist of rigid body (translation and rotation) and deformation components [22]. The majority of the STA is produced by the rigid movement rather than by its deformation. Deformation can be avoided through the use of the rigid plate with markers. So, only the STA rigid component was considered in our study. We utilised public ex-vivo STA data from [23] which provides the pelvic and femoral anatomical coordinate system (ACS) and STA characteristics of 12 markers in the femoral ACS during hip movement. M04 and M07 markers were selected to model the STA because their marker positions are mostly close to the cluster placements on the segment. A mathematical model was calculated based on a linear relationship between the measured STA and hip joint kinematics using least square method [15, 19, 24]. The same modelled noise was applied to all markers on the same rigid plate. We computed the CoR with the noisy marker data and calculated the RMSEs for all functional methods.

## Results

**Fig 2:**
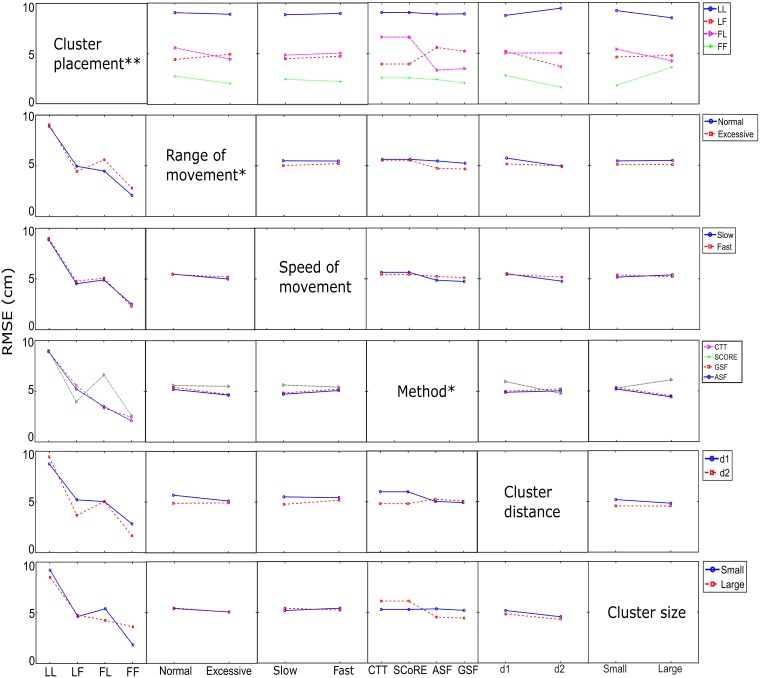
General linear model ANOVA effect matrix applied to the CoR estimation error (RMSE). Results demonstrated that the cluster placement, range of movement, functional methods have significant influence on the results. Significant effects at *α* < 0.05 are denoted with * and *α* < 0.001 with **.

Results from the general linear model ANOVA (Fig 2) showed that the factors, such as speed of movement, cluster size and cluster distance, had no significant influence on the CoR estimation whereas the cluster placement, the range of movement and functional methods were significant factors. The cluster placement was the most significant factor that affected the CoR estimation. The CoR estimation using front marker clusters on the proximal and distal segments (FF) were significantly closer to the CoR reference than other cluster combinations. The best CoR estimation with a mean RMSE of 4.7 mm was achieved using the front clusters and ASF method.

Fig 2 showed that factor impacts on the two types of methods were different. Compared to the transformation techniques, the sphere fitting methods were more sensitive to the range of movement and speed of movement. Slow and excessive movement was significantly closer to the CoR reference than other motion patterns when using the ASF and GSF methods. The CTT and SCoRE achieved more accurate functional CoR when the distal cluster was placed closer to the joint. The ASF and GSF methods, by contrast, were less affected by the cluster distance. Moreover, the accuracy of CoR estimation with sphere fitting methods was improved when using large cluster while the factor had opposite influence on the CTT and SCoRE methods.

**Fig 3:**
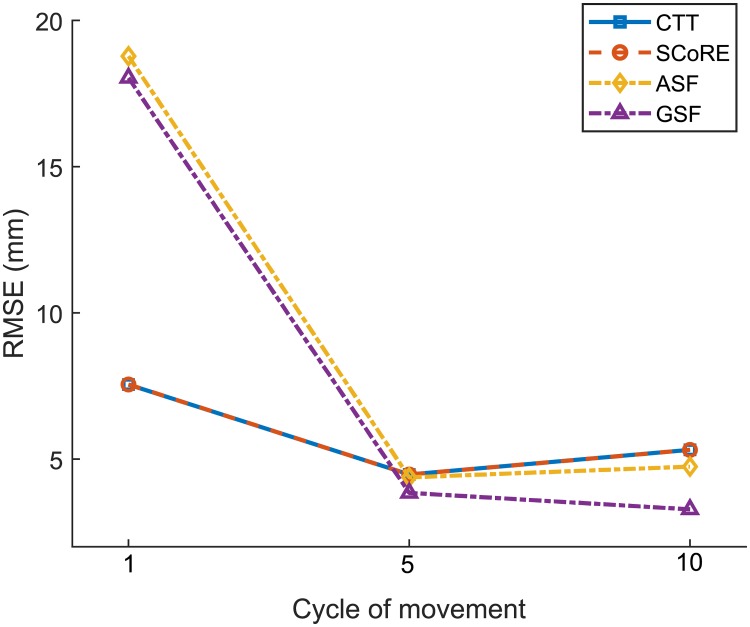
CoR localisation error (RMSE) for functional methods with varying the cycle of movement. Results showed that the accuracy of CoR estimate was significantly improved by increasing the cycle of movement from 1 and 5. The data is relative to fast and normal movement.

Functional methods were not sensitive to the speed of movement. As five cycles of movement were used for computing CoR location in our study, the number of data samples were different in slow and fast movement trials, which does not significantly affect the accuracy of CoR localisation. We analysed the estimated RMSE associated with the cycle of movement as shown in Fig 3. The RMSE was reduced significantly by increasing the cycle of movement from 1 to 5. Sphere fitting algorithms were more sensitive to the number of cycles than the transformation techniques. No significant improvement was found using more than five cycles of movement.

**Fig 4:**
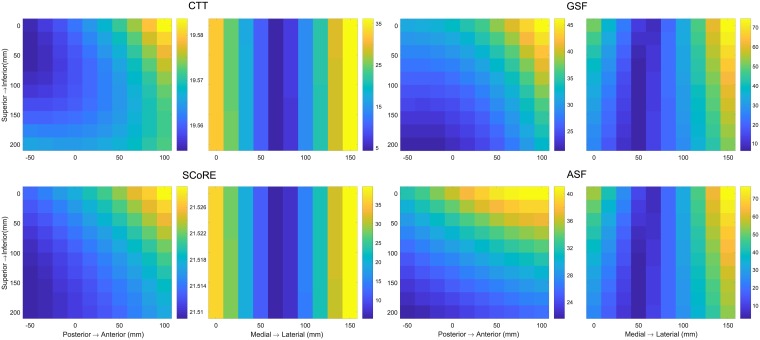
Error distribution of the proximal coordinate origin varying in the frontal and transverse planes when four different functional methods were applied. The origin of the proximal coordinate system was varied with increment steps of 10 mm and ranged from 0 to 150 mm along medial-lateral axis, from -50 to 100 mm along the posterior-anterior axis and from 0 to 2000 along the inferior-superior axis.

The location of the proximal coordinate had an important influence on the CoR estimation as shown in Fig 4. The RMSE results varied from 4.35 to 36.38 mm in the transformation algorithms and changed from 2.27 to 81.42 mm using the sphere fitting methods. There was a significantly strong positive correlation between the displacement of the proximal coordinate origin to the joint centre along the medial-lateral axis and the accuracy of CoR estimation (Table 2).

**Table 2:** Correlation coefficients of distance of proximal and distal coordinate origin from the joint centre against CoR errors.

	Proximal coordinate system			Distal coordinate system		
	Posterior-anterior	Medial-lateral	Inferior-superior	Posterior-anterior	Medial-lateral	Inferior-superior
CTT	5.42e-04	0.94*	-1.22e-04	6.21e-03	0.53*	-0.08
SCoRE	3.28e-04	0.92*	-2.24e-04	0.01	0.54*	-0.08
ASF	0.12*	0.92*	-0.22*	0.90*	0.96*	-0.41*
GSF	0.23*	0.9*	-0.21*	0.86*	0.91*	-0.43*

* denotes that the correlation is significant (*p* < 0.05).

**Fig 5:**
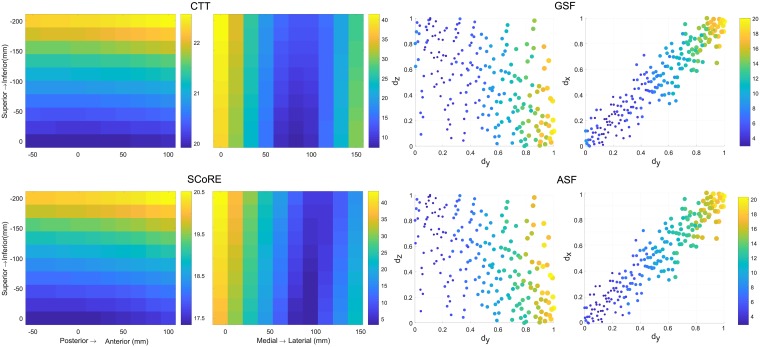
Contribution of the distal marker placement to the CoR estimation. Average root mean square error (RMSE) distribution of the distal coordinate origin varied in the frontal and transverse planes were computed for the CTT and SCoRE while the correlation between the centroid of markers and RMSE was analysed for the GSF and ASF methods.

The effect of the distal coordinate variation on the CoR errors when the CTT and SCoRE methods used was shown in Fig 5. The medial-lateral distance of the origin to the joint centre was significantly related to the CoR errors and the correlation coefficient showed there was a moderate positive relationship (Table 2). The contribution of the centroid of selected markers to the CoR estimation was analysed for the ASF and GSF methods as shown in Fig 5. A significantly strong positive relationship was observed between the medial-lateral and posterior-anterior displacement and the RMSEs while a significantly weak relationship was found for the superior-inferior distance to the joint centre.

**Fig 6:**
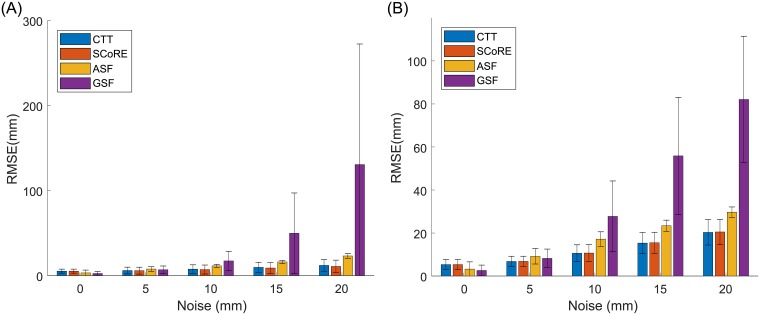
CoR estimate errors (RMSE) when varied amplitudes of random Gaussian noise. The random Gaussian noise was applied to the proximal (A) and distal (B) markers respectively. The mean changes associated with the CTT and SCoRE algorithms are smaller than the mean changes from the sphere fitting methods. Each error bar represents one standard deviation. The data is relative to “fast and normal” motion pattern.

Introducing random Gaussian noise caused large increases in CoR errors. The sphere fitting algorithms, particularly the GSF method, were more sensitive to the applied noise compared to the CTT and SCoRE (Fig 6). When 20 mm of noise was applied, the mean errors associated with the GSF and CTT increased by 51.67 ± 32.54 mm and 23.01 ± 9.15 mm respectively. There was a significant difference (*p* < 0.001) between the ability of the transformation and sphere fitting algorithms to reject noise with the quasi-planar movement.

**Fig 7:**
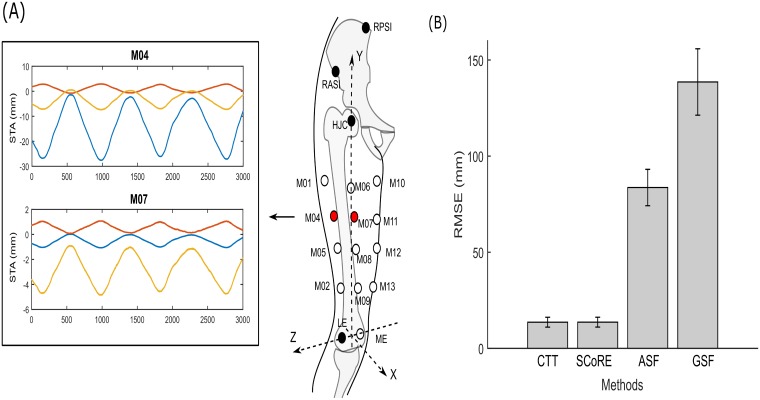
The CoR estimate results when noise modelled from realistic soft tissue artefacts (STA) was introduced. (A) The realistic STA was modelled based on a linear relationship of hip joint angle in femoral anatomical coordinate system (ACS) of the mechanical linkage for “fast and normal” motion. The ex-vivo data from [23] was used. The dataset provides the pelvic and femoral ACS and STA characteristics of 12 markers in the femoral ACS during hip movement. M04 and M07 markers were selected to model the STA because their marker positions are mostly close to the cluster placements on the segment. A mathematical model was used based on a linear relationship between the measured STA and hip joint kinematics and model coefficients of M04 and M07 markers were calculated using least square methods respectively. (B) Errors of CoR localisation with all four algorithms when the modelled STA applied to the distal markers.

STAs modelled from ex-vivo data (Fig 7A) were added to the markers on the lateral and front distal clusters. Larger STA was generated in the marker on the lateral side of the thigh (M04) compared to the front marker (M07). When the STA was applied, the transformation algorithms achieved more accurate CoR location (< 14 mm) than the ASF and GSF methods. The GSF was most sensitive to the introduced STA with a mean RMSE of 138.56 ± 17.26 mm.

## Discussion and conclusion

The accuracy of CoR estimation is sensitive to the implementation of functional methods [3, 7, 12, 25]. In this study, we used a mechanical linkage to investigate the performance of four functional methods that determine the CoR during a quasi-planar movement. The use of a mechanical linkage provides a good tool for giving insight to variation in implementation of functional methods and their effect on the accuracy of CoR estimation [5, 10]. It would help to establish an upper limit on the accuracy of functional methods with well-defined joint parameters. The simulation results provide guidelines for the use of functional methods in human subjects with restricted planar movement. Our results showed that the precision of the CoR localisation was sensitive to a number of experimental and analytical factors, particularly the location of local coordinate systems.

Four algorithms were selected and submitted to comparative analysis. The CTT and SCoRE methods exhibited similar performances in terms of accuracy with a mean error of 5.33 ± 2.38 mm. The sphere fitting methods yielded errors of 2.58 ± 2.49 and 3.22 ± 3.37 mm respectively for the GSF and ASF. The results are comparable with previous studies [10, 16]. The CoR error with the transformation techniques is larger than the results reported in [16] while the sphere fitting methods obtained a better estimation compared to [10]. The increased error in the CTT and SCoRE might be caused by the propagation of the stereophotogrammetric error when markers are close to each other on a rigid plate [20]. Piazza et al. [10] reported that a sphere fitting algorithm was sensitive to the range of motion whereas our results also showed that increasing the range of motion reduced the error significantly statistically in sphere fitting methods (Fig 2). No significant difference in the mean CoR errors was found between quasi-single planar motions with varying amplitudes when a transformation algorithm was used [16] which is also consistent with our results.

Functional methods assume a fixed CoR during the movement and mathematically estimate the position of the CoR by minimising the objective function of the whole marker dataset [3]. Consistent with Camomilla et al. [5], we found that the location of distal marker cluster is critical for the performance of the functional methods during quasi-planar motion. Fig 5 showed that the CoR error was significantly affected by the distal marker cluster parameters. The centroid of the marker cluster should be placed as close as possible to the joint centre. For sphere fitting algorithms, reducing the cluster dimension increased the CoR errors as the factor amplifies the influence of the stereophotogrammetric error on the markers [20]. The effect of proximal coordinate origin was firstly analysed in our study. The CoR error increased when increasing the distance of the coordinate from the joint centre at the medial-lateral axis (Fig 4). The proximal cluster should be placed as close as possible to the joint centre along the medial-lateral axis but as far as possible from the joint along the inferior-superior axis. In any case, our results demonstrated that marker clusters should be located close to the joint centre from the medial-lateral direction in order to compensate for the lack of motion information in that plane.

The number of data samples for optimal functional calibration should be great than 500 at a sample rate of 60 Hz (execution time ≈ 10s) [5]. The increase of data sample number from 240 to 500 resulted in a 40% reduction of estimation errors. Fig 3 showed that at least 5 cycles of movement should be executed (≈ 10s), which is consistent with the results of the previous study. Sphere fitting methods were more sensitive to the number of movement cycles. CTT and SCoRE would be preferred when the subject has difficulty to repeat the movement.

We evaluated the performance of the algorithms with random Gaussian noise and modelled STA as shown in Figs 6 and 7. The results have shown that functional methods have worse noise rejection performance when the movement was mainly constrained in a single plane. With presence of both types of noise, sphere fitting algorithms are more sensitive to the applied noise compared to the CTT and SCoRE. The finding is consistent with results of [16]. The ASF results in smaller RMSE than the GSF as the bias compensation procedure may reduce the influence of noise on algorithm performance [18].

Recent human in-vivo studies investigated the accuracy of HJC localisation using functional methods against medical imaging techniques and compared the HJC estimate error of predictive techniques and functional methods [9, 11, 12, 14]. All study results supported that the Harrington method is the best predictive method [9, 11, 13, 14]. However, the results about functional methods are different between studies. Sangeux et al. [11] reported that the sphere fitting methods performed best in functional techniques with an accuracy of approximately 15 mm. Similar results were also demonstrated in [13, 14]. On the other hand, Fiorentino et al. [9] reported an average accuracy of 11.1 and 10.8 mm for Schwartz transformation technique and SCoRE method respectively. Despite medical image technique used in the two studies [9, 11], the main difference between the experiment settings was the type of marker cluster: skin [11] and rigid marker cluster [9]. The STA plays a major role in causing inaccuracies in the HJC estimation. Results from simulation study of Ehrig et al. [3] showed that functional methods achieved better CoR estimation when Gaussian noise (standard deviation 0.1cm) were applied to all markers on the segment than when identically distributed Gaussian noise (standard deviation 0.1cm) was applied to each marker. The results imply that the use of rigid marker cluster may improve the accuracy of functional methods by eliminating the marker cluster deformation, especially for the CTT and SCoRE. Further study is needed to investigate the effect of individual markers and marker clusters on HJC estimation in human subjects.

Functional methods have shown better overall agreement with the joint centre reference than predictive methods on healthy adults [9, 11, 12]. Studies that investigated the accuracy of both the predictive regression methods and functional methods on children with cerebral palsy [13, 26] reported that the Harrington equation achieved best agreement with medical image approaches in the HJC prediction. The difference in results obtained between the adults [11] and children [13] could be related to the shorter thigh segment in children. Closer marker distance might amplify the influence of stereophotogrammetric errors in the HJC estimation. Less muscle tone may also be a significant factor of the HJC errors [14]. The Harrington and functional methods are most appropriate for clinical use [9]. The Harrington method is preferred when applied to patients with motor control impairment, such as patients with cerebral palsy or hip osteoarthritis [14]. Current functional method implementation is more suited for healthy adults.

Current implementation of functional methods has its limitation in human study when hip motion was performed in reduced planes [5, 7, 14]. When a subject performed a thigh movement incorporating the sagittal and frontal plane motion, the largest error in CoR estimation was observed in the medial-lateral direction [7] where the marker motion was unavailable. Our results have shown that the placement of proximal coordinate is crucial for the performance of functional algorithms during restricted plane movements and the proximal coordinate origin compensates for the lack of information in the plane. This may explain the increased CoR errors due to a limited motion where the pelvis coordinate origin was determined as the midpoint of anterior/posterior pelvis markers [5, 7, 27]. An optimal proximal coordinate location could improve the CoR estimate accuracy during restricted plane movements. Functional methods may obtained better HJC estimation on subjects with motion dysfunction when a optimised implementation is applied.

In general, according to the results in our study, it is possible to determine CoR when quasi-planar movement occurs. Factors that may significantly affect the accuracy of CoR location are summarised in Table 3. Our results complied with guidelines proposed by Camomilla et al. [5] that centroid of markers located as close as possible to the hip and markers located at the possible distance from each other. Moreover, we would like to propose additional factors that need to be considered for practical implementation. Firstly, the proximal marker cluster should be placed as close as possible from the joint centre along the medial-lateral direction whilst as far as possible along the inferior-superior axis. Secondly, the CTT and SCoRE algorithms are superior to the sphere fitting methods when the noise was applied. Finally, the range of movement is not a significant factor when using the transformation methods. After an optimal protocol incorporating the above-mentioned factors is set-up, additional work is needed to evaluate the protocol in a clinical environment.

**Table 3:** Summary of significant factors for functional methods on CoR estimation.

	Significant factors
CTT and SCoRE	Cluster placement is the most significant factor: there is a strong/moderate positive correlation between the displacement of the proximal/distal coordinate origin to the joint centre along the medial-lateral axis and the CoR location errors. The methods achieve more accurate CoR localisation when the distal cluster is placed closer to the joint centre. The algorithms are more robust against the simulated STA.
ASF and GSF	Cluster placement is the most significant factor: A strong positive correlation was found between the medial-lateral displacement of proximal coordinate origin to the joint centre and the CoR errors. The centroid distal marker set should be as close as possible to the joint centre along the medial-lateral direction. The algorithms are more affected by the range of movement and speed of movement. The accuracy of CoR estimation was improved when using a larger cluster.
